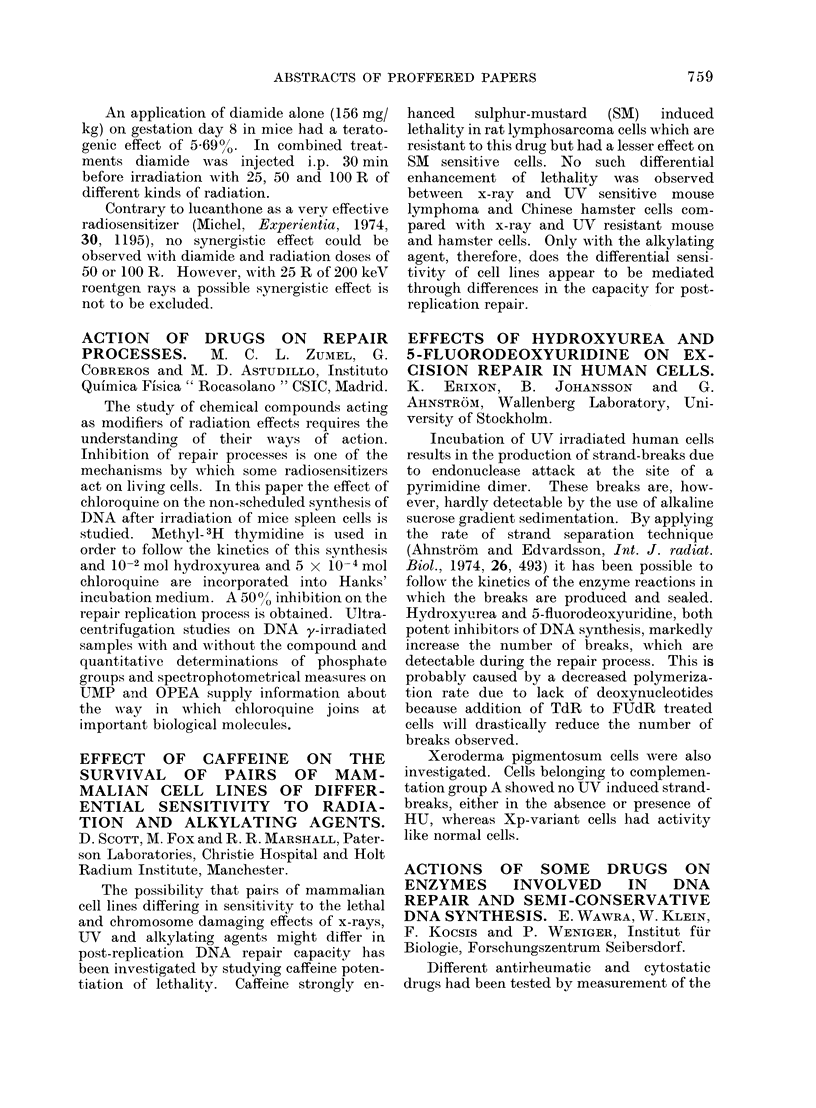# Proceedings: Action of drugs on repair processes.

**DOI:** 10.1038/bjc.1975.315

**Published:** 1975-12

**Authors:** M. C. Zumel, G. Cobreros, M. D. Astudillo


**ACTION OF DRUGS ON REPAIR PROCESSES.** M. C. L. Zumel, G. Cobreros and M. D. Astudillo, Instituto Química Física " Rocasolano " CSIC, Madrid.

The study of chemical compounds acting as modifiers of radiation effects requires the understanding of their ways of action. Inhibition of repair processes is one of the mechanisms by which some radiosensitizers act on living cells. In this paper the effect of chloroquine on the non-scheduled synthesis of DNA after irradiation of mice spleen cells is studied. Methyl-$^3$H thymidine is used in order to follow the kinetics of this synthesis and $10^{-2}$ mol hydroxyurea and $5 \times 10^{-4}$ mol chloroquine are incorporated into Hanks' incubation medium. A 50% inhibition on the repair replication process is obtained. Ultracentrifugation studies on DNA $\gamma$-irradiated samples with and without the compound and quantitative determinations of phosphate groups and spectrophotometrical measures on UMP and OPEA supply information about the way in which chloroquine joins at important biological molecules.